# Endovascular Coiling Versus Neurosurgical Clipping for Aneurysmal Subarachnoid Hemorrhage: A Systematic Review and Meta-analysis

**DOI:** 10.7759/cureus.4320

**Published:** 2019-03-26

**Authors:** Syed Ijlal Ahmed, Gohar Javed, Syeda Beenish Bareeqa, Syeda Sana Samar, Ali Shah, Arwa Giani, Zainab Aziz, Abeer Tasleem, Syed Hasham Humayun

**Affiliations:** 1 Neurology, Liaquat National Hospital and Medical College, Karachi, PAK; 2 Neurosurgery, The Aga Khan University, Karachi, PAK; 3 Oncology, Jinnah Medical and Dental College, Karachi, PAK; 4 Internal Medicine, Jinnah Sindh Medical University, Karachi, PAK; 5 Surgery, Dow University of Health Sciences, Karachi, PAK; 6 Miscellaneous, Ziauddin Medical University, Karachi, PAK; 7 Neurology, Ziauddin Medical University, Karachi, PAK; 8 Miscellaneous, Jinnah Medical and Dental College, Karachi, PAK

**Keywords:** neurosurgery, sub arachnoid hemorrhage, aneurysm clip, endovascular, coiling

## Abstract

Background

Aneurysmal subarachnoid hemorrhage is a frequently devastating condition with a reported incidence of between 10 and 15 people per 100,000 in the United States. Currently, according to the best of our knowledge, there are not enough meta-analyses available in the medical literature of the last five years which compare the risks and benefits of endovascular coiling with neurosurgical clipping.

Methods

Twenty-two studies were selected out of the short-listed studies. The studies were selected on the basis of relevance to the topic, sample size, sampling technique, and randomization. Data were analyzed on Revman software.

Results

Mortality was found to be significantly higher in the endovascular coiling group (odds ratio (OR): 1.17; confidence interval (CI): 95%, 1.04, 1.32). Re-bleeding was significantly higher in endovascular coiling (OR: 2.87; CI: 95%, 1.67, 4.93). Post-procedure complications were significantly higher in neurosurgical clipping compared to endovascular coiling (OR: 0.36; CI: 95%, 0.24, 0.56). Neurosurgical clipping was a 3.82 times better surgical technique in terms of re-bleeding (Z = 3.82, p = 0.0001). Neurosurgical clipping is a better technique requiring fewer re-treatments compared to endovascular coiling (OR: 4.64; CI: 95%, 2.31, 9.29). Endovascular coiling was found to be a better technique as it requires less rehabilitation compared to neurosurgical clipping (OR: 0.75; CI: 95%, 0.64,0.87).

Conclusion

Neurosurgical clipping provides better results in terms of mortality, re-bleeding, and re-treatments. Endovascular coiling is a better surgical technique in terms of post-operative complications, favorable outcomes, and rehabilitation.

## Introduction

Aneurysmal subarachnoid hemorrhage is a frequently devastating condition with a reported incidence between 10 and 15 people per 100,000 population in the United States [1]. Acute subarachnoid hemorrhage (SAH) is most commonly diagnosed with a non-contrast cranial computed tomography (CT) scan. Diagnostic lumbar puncture should be performed if the initial CT scan is negative. In the first 12 hours after SAH, the sensitivity of CT for SAH is 98% to 100%, declining to 93% at 24 hours [2] and to 57% to 85% six days after SAH [3]. Surgical clipping and endovascular coiling are the most commonly performed methods to reduce the rate of re-bleeding after SAH. In endovascular coiling, a micro-catheter is inserted into the femoral artery via an initial catheter. A platinum coil is attached to the microcatheter tip. When the microcatheter reaches the lumen of the aneurysm, an electrical current is used to separate the coil from the catheter. The coil induces thrombosis of the aneurysm and is left permanently in the aneurysm. Surgical clipping is done under general anesthesia and requires open surgery. The brain is gently retracted to visualize the aneurysm. A small clip is placed across the neck of the aneurysm to block the blood flow into it. Clips are made of titanium and remain on the artery permanently. An international subarachnoid trial study shows that surgical clippings have a better outcome at one year, in terms of survival free of disability [4].

The procedural complications of endovascular coiling are classified as ischemic (e.g., thrombo-embolic, spasm during treatment), hemorrhagic (e.g., aneurysm rupture), and technical (e.g., coil in the vessel and aortic dissection) complications [5].

The complications of neurosurgical clipping may include a hemorrhagic event, brain swelling (edema), infarction, hypotension, and cardiac arrhythmias [6].

Currently, according to the best of our knowledge, there are not enough meta-analyses available in the medical literature of the last five years which compare the risks and benefits of endovascular coiling with microsurgical clipping. Our study is an updated meta-analysis involving the most current studies to compare mortality, the re-bleeding rate, favorable outcomes, and the ability of each procedure to reach an appropriate conclusion.

## Materials and methods

Literature search strategy

We’ve conducted this meta-analysis in accordance with the Preferred Reporting Items for Systematic Reviews and Meta-Analysis (PRISMA) guidelines [7]. A detailed literature search was conducted by two independent authors using the keywords 'micro-vascular clipping', 'endovascular coiling', 'subarachnoid hemorrhage', 'ruptured aneurysm', and 'favorable outcome' to search Scopus, PubMed, Ovid Medline, Google Scholar, and Cochrane library databases. Relevant terms or synonyms other than keywords were utilized to conduct a comprehensive search in accordance with the pre-specified eligibility criteria. All the searched articles were exported and cited through Endnote. The search strategy was limited to medical literature in English, published from 2013 until present. In cases of unavailability of full text or incomplete data, the corresponding author was contacted. 

Eligibility criteria

The study types which have been included in our research were randomized controlled trials and both prospective and retrospective cohort studies. However, case reports, letters to the editor, commentaries, cross-sectional surveys, and documentaries were excluded but used only to bridge and link the outcomes of our study with past medical research for discussion. Moreover, studies in non-English literature; studies which assessed the outcome in pathologies other than intracranial aneurysm; interventions other than endovascular coiling or micro-vascular clipping (such as wrapping of aneurysm) used in a study; studies without definitive numbers or values; experimental animal trials; and studies with figurative or graphical results presentation without any particular numerical values were also excluded from this research. Two independent authors retrieved the required data in accordance with the mentioned eligibility criteria. Any disagreement was resolved by collaborative discussion.

Data collection

The studies were assessed and the data were extracted by two independent reviewers according to the PRISMA guidelines. Data were collected and compiled on a predefined evidence table. Articles were selected on the basis of relevance to the topic, sample size, sampling technique, and randomization. The collected data include author, year of publication, sample size, study design, mortality, re-bleeding, re-treatments, post-operative complications, the statistical results of the study (RR, CI, and p-value), favorable outcomes (Modified Rankin scale scores ranging from zero to two), and rehabilitation. Any disagreement was resolved with collaborative consensus.

Quality assessment and risk of bias

To assess the quality of extracted data, the Newcastle-Ottawa scale was used for cohort studies and the Jadad scale (also known as Oxford quality scoring system) was utilized for randomized controlled trials.

Data analysis and primary outcomes

The data was entered on Review Manager (version 5; The Nordic Cochrane Centre, Copenhagen) and was analyzed using a forest plot for visual estimation of meta-analysis. The test for heterogeneity was also done (p < 0.05). A fixed effect model with an inverse variance method was used to obtain the overall odds ratio estimates and the 95% confidence interval in order to assess the effect of endovascular coiling and neurosurgical clipping on mortality, re-bleeding, postoperative complications, favorable outcomes, retreatment, and rehabilitation in aneurysmal subarachnoid hemorrhage patients. A p-value of less than 0.05 was considered significant. 

## Results

Study selection

**Figure 1:**
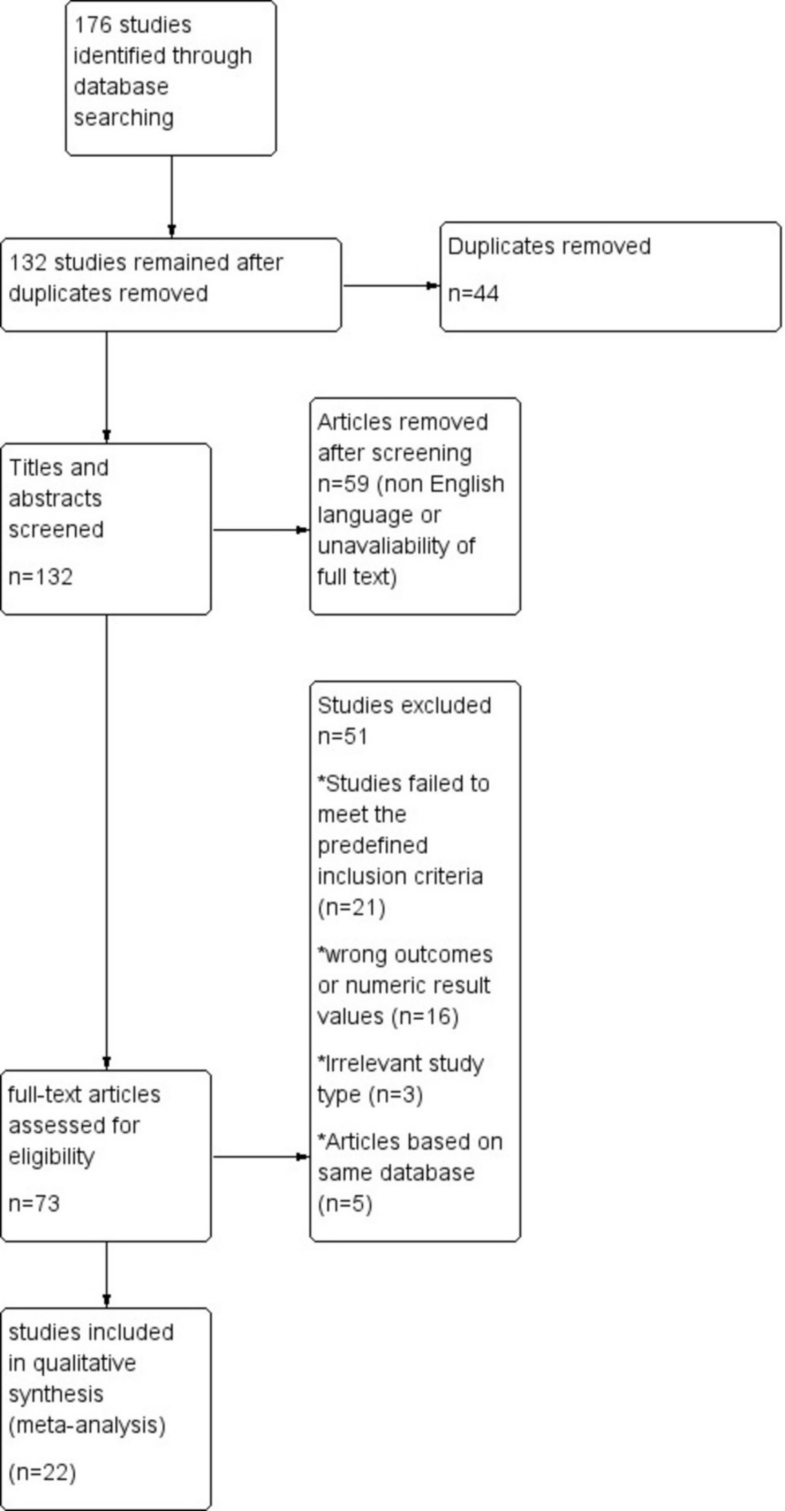
The flow diagram for data extraction strategy in accordance with PRISMA guidelines PRISMA: Preferred Reporting Items for Systematic Reviews and Meta-Analysis

We selected 176 articles (including cohort, case-control, and randomized trials, and reviews) on the basis of relevant titles and abstracts after a systematic review. After going through the abstracts of the selected articles, 44 duplicates were excluded from the selected pool. The remaining 132 articles were screened for the required information. Another pool of 59 articles was removed after the screening of titles and abstracts. Removal was on the basis of non-English language literature and the unavailability of the full text of articles. The rest of the 73 full-text articles were assessed in accordance with the eligibility criteria, out of which 22 articles were finalized for quantitative synthesis [8-29]. The Flow diagram for data extraction strategy in accordance with PRISMA guidelines [7] is given in Figure 1.

Study characteristics

The studies were compared on the basis of mortality, re-bleeds, post-operative complications, favorable outcomes, re-treatments, and rehabilitation. A total of 8,836 patients were included in the endovascular coiling group, while 7,294 patients were included in the neurosurgical clipping group.

Overall outcomes

Mortality was found to be significantly higher in the endovascular coiling group, i.e., 1,042 events in 3,973 patients in comparison to neurosurgical clipping, where mortality was found among 652 out of 3,309 patients (OR: 1.17; CI 95%, 1.04, 1.32). Neurosurgical clipping was found to be 2.57 times better than endovascular coiling in terms of mortality (Z = 2.57, p = 0.01).

Regarding re-bleeding, it was reported in 49 out of 1,570 patients in endovascular coiling and 17 out of 1,646 patients in neurosurgical clipping. Re-bleeding was significantly higher in endovascular coiling (OR: 2.87; CI 95%, 1.67, 4.93). Neurosurgical clipping was 3.82 times a better surgical technique in terms of re-bleeding (Z = 3.82, p = 0.0001).

Post-procedure complications were significantly higher in neurosurgical clipping compared to endovascular coiling. Post-procedure complications were reported in 54 out of 545 patients in endovascular coiling compared to neurosurgical clipping in 68 out of 270 patients (OR: 0.36; CI 95%, 0.24, 0.56). Endovascular coiling was found to be 4.35 times a better technique compared to neurosurgical clipping in terms of post-operative complications.

Favorable outcomes were more frequent in the endovascular coiling group than neurosurgical clipping (Z=3.04, p<0.002)

Thirty-nine out of 301 patients required re-treatment in the endovascular coiling group compared to 13 out of 339 patients in the neurosurgical clipping group (OR: 4.64; CI: 95%, 2.31, 9.29). Therefore, neurosurgical clipping is 4.33 times a better technique requiring fewer re-treatments compared to endovascular coiling (Z = 4.33, p < 0.0001). 

In endovascular coiling, rehabilitation was required in 556 out of 2,146 patients compared to 422 out of 1,336 patients in the neurosurgical clipping group (OR: 0.75; CI 95%, 0.64, 0.87). Endovascular coiling was found to be 3.79 times a better technique as it requires less rehabilitation compared to neurosurgical clipping.

**Figure 2:**
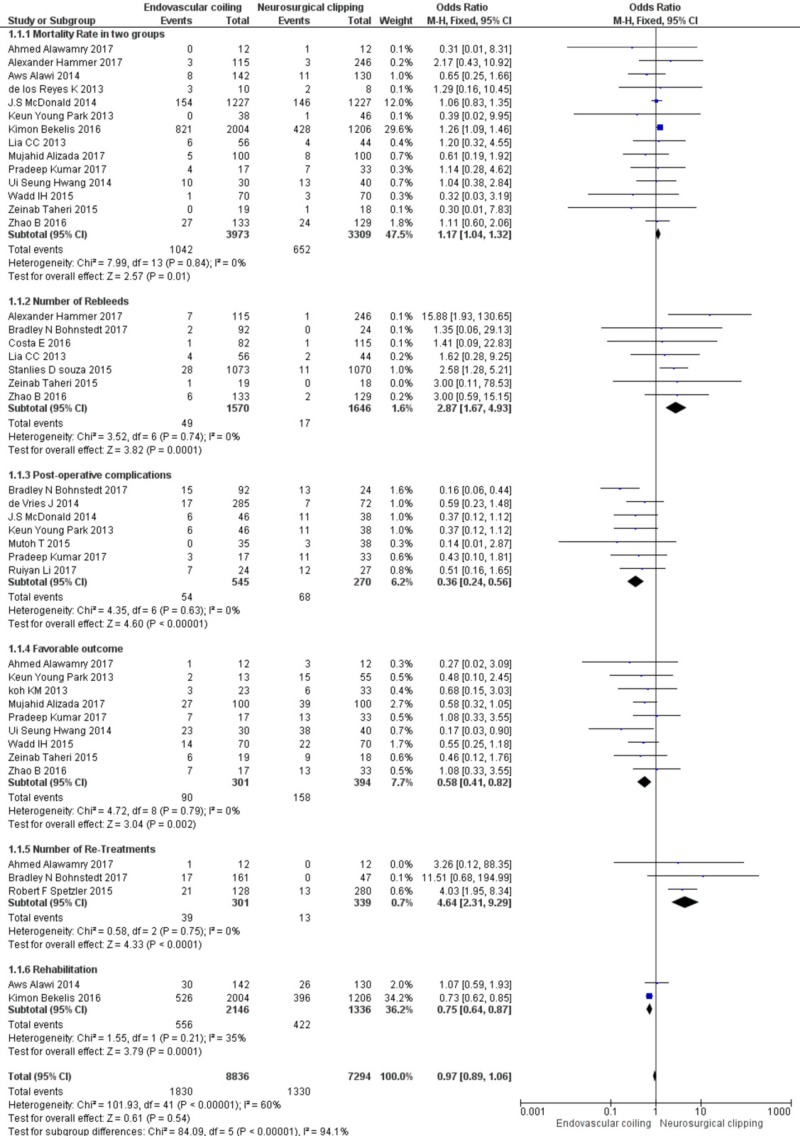
The detailed meta-analysis on endovascular coiling versus neurosurgical clipping

The overall effect was found to be Z = 0.61 (p = 0.54). The detailed meta-analysis on endovascular coiling versus neurosurgical clipping is shown in Figure 2.

**Figure 3:**
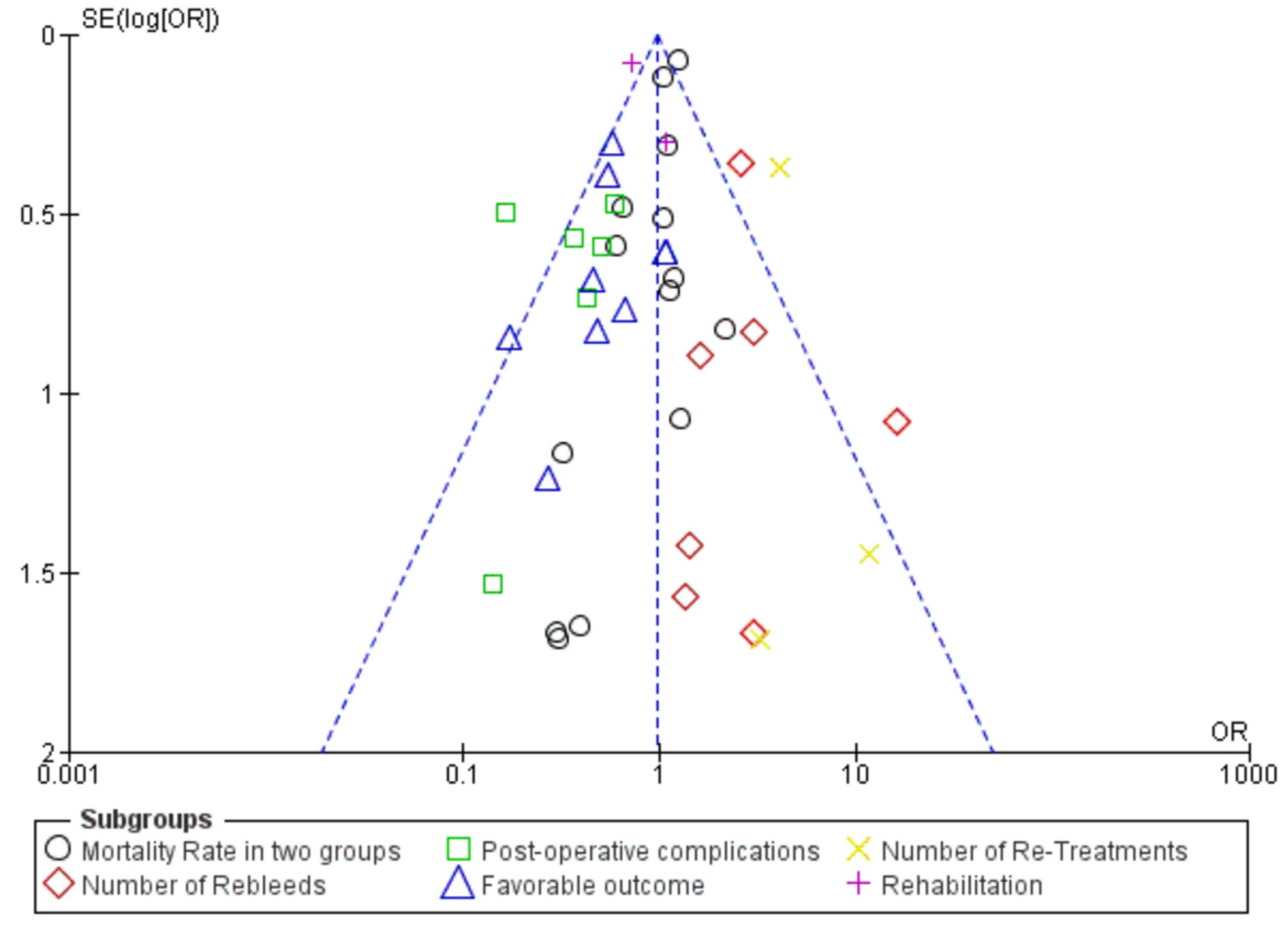
Figure shows funnel plot

The heterogeneity test was also performed, and the funnel plot is shown in Figure 3.

## Discussion

Subarachnoid hemorrhage is a life-threatening condition that occurs in about 1 per 10,000 people per year. There are growing concerns regarding which of the following treatment procedures, endovascular coiling (EVC) or neurosurgical clipping (NSC), yields the maximum observed clinical benefits for the patients. Therefore, this research was conducted to determine the relatively better method for the treatment of SAH. From our research, we concluded that neurosurgical clipping (NSC) yielded better results in terms of mortality, re-bleeding, and retreatment; whereas, endovascular coiling (EVC) had better results in terms of postoperative complications, favorable outcomes, and rehabilitation.

One study in elderly patients demonstrated that the number of patients independent after endovascular coiling was greater as compared to NSC. The location of the ruptured aneurysm had a significant impact on the choice of procedure. Hence the research suggests that EVC should be favored for intracranial aneurysm (ICA) and posterior communicating (PCOM) ruptured aneurysms, whereas for middle cerebral artery (MCA) ruptured aneurysm, NSC could be the treatment of choice. Furthermore, the one-year mortality rate was also higher for NSC. However, in accordance with our research, this article shows that the frequency of postoperative complications like epilepsy, infectious complications, pulmonary complications was relatively less in the patients treated with EVC [30].

Exclusion of bias

To assess publication bias in this meta-analysis, a visual interpretation of funnel plot symmetry and search of grey literature (like dissertations, conference proceedings, theses, and technical reports) was conducted by two independent reviewers. No publication bias was found in this study.

Limitations

Due to the unavailability of a language translator, our data is restricted to English-language literature only, which might have limited the inclusion of a significant amount of knowledge in our research. Secondly, due to the unavailability of skilled statisticians in meta-regression, the assessment of publication bias was limited to visual analysis of funnel plot symmetry and searching of grey literature.

## Conclusions

On the basis of this meta-analysis, we conclude that neurosurgical clipping provides better results in terms of mortality, re-bleeding, and re-treatments. Endovascular coiling is a better surgical technique in terms of post-operative complications, favorable outcomes (MRS scores ranging zero to two), and rehabilitation.
